# H2RM: A Hybrid Rough Set Reasoning Model for Prediction and Management of Diabetes Mellitus

**DOI:** 10.3390/s150715921

**Published:** 2015-07-03

**Authors:** Rahman Ali, Jamil Hussain, Muhammad Hameed Siddiqi, Maqbool Hussain, Sungyoung Lee

**Affiliations:** Department of Computer Engineering, Kyung Hee University, Seocheon-dong, Giheung-gu Yongin-si, Gyeonggi-do 446-701, Korea; E-Mails: rahmanali@oslab.khu.ac.kr (R.A.); jamil@oslab.khu.ac.kr (J.H.); siddiqi@oslab.khu.ac.kr (M.H.S.); maqbool.hussain@oslab.khu.ac.kr (M.H.)

**Keywords:** reasoning, H2RM, rough set theory, rules mining, RBR, prediction, classification, regression, diabetes mellitus, trend analysis

## Abstract

Diabetes is a chronic disease characterized by high blood glucose level that results either from a deficiency of insulin produced by the body, or the body’s resistance to the effects of insulin. Accurate and precise reasoning and prediction models greatly help physicians to improve diagnosis, prognosis and treatment procedures of different diseases. Though numerous models have been proposed to solve issues of diagnosis and management of diabetes, they have the following drawbacks: (1) restricted one type of diabetes; (2) lack understandability and explanatory power of the techniques and decision; (3) limited either to prediction purpose or management over the structured contents; and (4) lack competence for dimensionality and vagueness of patient’s data. To overcome these issues, this paper proposes a novel hybrid rough set reasoning model (H2RM) that resolves problems of inaccurate prediction and management of type-1 diabetes mellitus (T1DM) and type-2 diabetes mellitus (T2DM). For verification of the proposed model, experimental data from fifty patients, acquired from a local hospital in semi-structured format, is used. First, the data is transformed into structured format and then used for mining prediction rules. Rough set theory (RST) based techniques and algorithms are used to mine the prediction rules. During the online execution phase of the model, these rules are used to predict T1DM and T2DM for new patients. Furthermore, the proposed model assists physicians to manage diabetes using knowledge extracted from online diabetes guidelines. Correlation-based trend analysis techniques are used to manage diabetic observations. Experimental results demonstrate that the proposed model outperforms the existing methods with 95.9% average and balanced accuracies.

## 1. Introduction

Diabetes mellitus (DM) is a chronic disease, which is characterized by hyperglycemia that results from absolute or relative deficiency of insulin. It has affected over 200 million individuals worldwide [1]. According to World Health Organization (WHO) report, back in 2004, the number of diabetic patients will increase to 366 million by 2030 [2]. However, a recent report of the International Diabetes Federation has estimated this number up to 552 million by 2030 [3]. Similarly, “the number of Americans with diagnosed diabetes is projected to increase 165%, from 11 million in 2000 to 29 million in 2050” [1]. Many factors results into diabetes, but the most common are hereditary, inflammation, diet and environment [4]. Diabetes was declared a global epidemic by World Health Organization (WHO) because of its rapidly increasing incidence. These days, multidisciplinary studies are intended to first predict diabetes and then control it with treatment plan procedures. This has become one of the important research areas worldwide.

In medical diagnosis, it is quite difficult for physicians to make a diagnosis decision by evaluating the current conditions of a patient without referring to previous decisions with similar symptoms. For this reason, a number of clinical decision support systems (CDSS) [5,6,7,8,9] have been developed that assist physicians in their decisions [10]. Such systems have widely been applied for diagnosis, prediction, classification and risk forecasting of different diseases from electronic medical record (EMR) data. The area of risk forecasting of type-2 diabetes has been explored from EMR data with the use of machine learning techniques, such as Gaussian Naïve Bayes, Logistic Regression, K-nearest neighbor, classification and regression tree (CART), Random Forests and support vector machine (SVM) [2]. Ensemble of SVM and back-propagation neural networks (BP NN) is used over Pima Indian dataset to predict presence of diabetes [3] with improved accuracy. Stahl [4] has proposed a Linear and Bayesian Ensemble Modeling technique to predict glucose level in diabetes mellitus (DM) patient data. They evaluated their model with 47 patients’ data and validated with 12 datasets. Similarly, a prototype diabetic decision support system, based on a multi-layer perceptron neural network model has been developed [5] that predicts psychosocial well-being behavior, such as depression, anxiety, energy and positive well-being of patients. In this system, patient’s biological or biographical variables, such as age, gender, weight and fasting plasma glucose are used as input predictors. In the literature [6], an architecture of multi-stage DM prediction system, based on fuzzy logic, neural network and case based reasoning (CBR) is proposed that uses two stages for prediction. In the first stage, base classifiers are used, whose results are forwarded to the second level, which uses a rule-based reasoner (RBR) for refinement of the initial results. Chen and Tan [7] have proposed a prediction model for T2DM. They used fisher linear discriminate analysis (FLDA), support vector machine (SVM) and decision tree (DT) algorithms for constructing prediction models. These models are based on several elements in blood and chemometrics of the patients. The elements considered include: lithium, zinc, chromium, copper, iron, manganese, nickel and vanadium. They constructed ensemble classifiers and validated the best ones on independent test data. Predicted results were compared with those of the real clinical diagnostics on the same subjects. According to the results, they claim that almost all classifiers produce similar performance, which implies that these elements can serve best as a valuable tool for diagnosing diabetes type-2. Sood *et al.* [8] have performed comparative analysis on various classification algorithms over electronic health records (HER) data of T2DM patients. They used Logistic Regression, Naïve Bayes, k-NN, Random Forest, Gradient Boosting Machine, SVM and Ensemble methods. They constructed different Ensemble models for prediction, but their findings conclude that the mean of Random Forest and Gradient Boosting method can perform best, both on training and testing datasets. Prediction models for type-1 diabetes mellitus in juvenile subjects are developed [9] using neural networks, decision trees and ensembles of both of these classifiers. For ensemble techniques, they used bagging and random forest algorithms. In a recent study [10], boosting ensemble classifier model is used for prediction of T1DM and T2DM. This model uses random committee classifier as the base classifier and achieves 81% prediction accuracy.

Apart from the listed literature, rough set theory, a powerful mathematical tool [11,12], has successfully been applied in medical diagnosis and prediction. For example, toxicity predictions [13], medical expert system rules creation [14], pneumonia patient’s death prediction [15], and chest pain prediction [16]. Other applications of RST includes: patients satisfaction analysis [17], extensions in fundamental rough set theory [12], rough set-based case studies and software implementation [18], rough set-based framework for medical diagnosis systems [19], and rough set-based identification of medical practice after total hip arthroplasty [20].

For the purpose of diabetes prediction, RST is applied over Pima Indian dataset [21] that has produced 75% accuracy [22]. Pima Indian dataset has successfully be used in a number of studies, for example, development of a java-based T2DM prediction tool [23], diabetes data analysis and prediction model [24], and decision tree based diabetes mellitus prediction model [25]. Similarly, for investigating relationship between psychosocial variables in Kuwaiti diabetic children, RST builds a classifier function that correctly classifies patients [26]. RS-based data analysis of the genetic data of children with T1DM is performed in [27] for rules extraction and prediction of children with genetic susceptibility to T1DM. This system recommends pre-diabetes therapy to patient if they are susceptible to type-1 diabetes. Similar study for children with T1DM can also be read in [28,29].

Apart from prediction of diabetes into its types, either using traditional learning methods or rough set techniques, future trend analysis and risk prediction is an important research area and have been approached with various techniques. For example, T2DM risk prediction using multivariate regression model [30] and T2DM prediction, in elderly Spanish population, having high cardiovascular risk, using multivariate cox regression model [31]. Other risk prediction models for type-2 diabetes can be read from the systematic review [32]. A multivariate logistic regression equation has been developed and validated with non-diabetic Egyptian subjects data. This equation has 62% sensitivity, 96% specificity, and 63% positive predictive score [33].

To our knowledge, the models and methods proposed in the literature have a number of limitations, which include: (1) neither of the study presents classification model for both T1DM and T2DM, but restricted either to one or the other type; (2) less explanation power in terms of understandable rules; (3) restricted either to prediction tasks or future trend analysis tasks over the structured contents; (4) lacking competence for handling dimensionality, inconsistencies and vagueness issues of clinical data; and (5) dependency on the assumptions of statistical techniques.

To overcome these limitations, this study proposes a hybrid rough set reasoning model that uses experiential and domain knowledge to accurately predict diabetes types and analyze future trends for potential risks. The experiential knowledge is obtained from patients’ clinical charts using manual parsing, while domain knowledge is translated from online diabetes guidelines with the help of domain experts. The experiential knowledge is first mined for prediction rules using rough set techniques, which are then used for predicting diabetes types. Domain knowledge is used to assist physicians in predicting future trend of risky observations and enabling them for prognosis services. Contributions of the study can be summarized as follows.

- Extraction of experiential knowledge, from unstructured patient’s clinical charts to a structured dataset.
- Translation of unstructured diabetes guidelines to domain knowledge for assisting physicians in future trend analysis and predicting potential risks.
- Mining understandable and self-explanatory prediction rules from high dimensional, inconsistent, and vague clinical data using powerful rough set theory.
- Generating integrated services for predicting types of diabetes (*i.e.*, T1DM, T2DM) and future trend analysis of risky observations that support physicians in prognosis services.

The rest of the paper is structured as follows: Section 2 proposes H2RM model and its working mode. Section 3 describes methodology of knowledge acquisition from clinical charts and online guidelines using rough set techniques and domain expert’s knowledge, respectively. This section also describes the online knowledge execution process for prediction and future trends analysis of diabetes types. Section 4 focuses on experiments, results and evaluation tasks, while Section 5 concludes the work done with future directions.

## 2. Proposed Hybrid Rough Sets Reasoning Model

**Figure 1:**
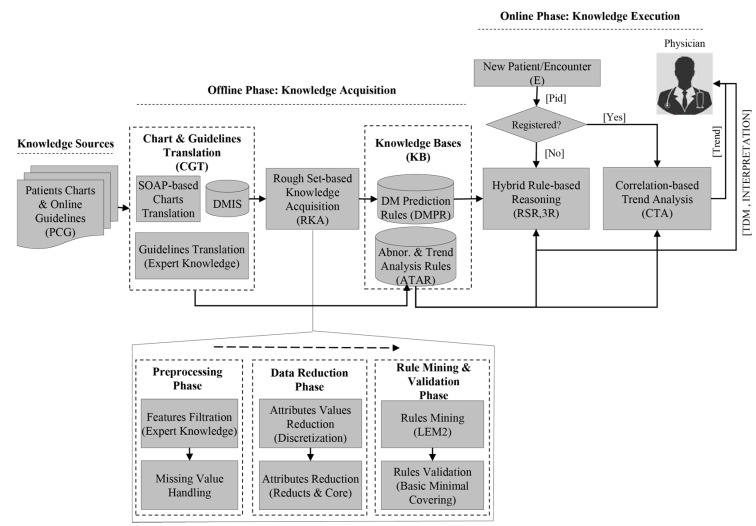
Hybrid rough set reasoning model for prediction and management of diabetes mellitus.

This work proposes and designs a hybrid model for prediction and management of T1DM and T2DM. A set of RS prediction rules are mined from 50 diabetes patients’ data that is acquired from a local hospital. The data is recorded during 2008–2011 in clinical chart format that follows subjective, objective, assessment, and plan (SOAP)-based protocol. For management, including finding abnormalities identification and predicting future trends, online diabetes guidelines are translated to simple reference range rules. The future trends analysis assists physicians in their diagnosis and prognosis processes. To support these functionalities, the proposed model is provided with the following components: patients charts and online guidelines (PCG) as knowledge sources, charts and guidelines translation (CGT), rough set-based knowledge acquisition (RKA), knowledge bases (KBs), hybrid rule-based reasoning (HRBR) and correlation-based trend analysis (CTA). Figure 1 shows the abstract view of the integrated model.

The order followed by the proposed model is: *PCG + CGT + RKA + KBs + HRBR + CTA*. Abstractly, this model can be represented as a sextuple: *PCG*, *CGT*, *RKA*, *KBs*, *HRBR*, and *CTA*, where:

- ***PCG*** *(patients charts and online guidelines*): Set of patients clinical charts, which are recorded by physicians during the patients visits to hospital, and online diabetes guidelines for managing patients abnormalities in observations and trend analysis. These constitute knowledge sources for the diabetes prediction and management.
- ***CGT*** *(charts and guidelines translation):* Set of methods and procedures used to translate clinical charts and online guidelines to structured data format and reference range rules, respectively. For charts, SOAP-based protocol is used to transform data into structured dataset, while for guidelines translation expert knowledge is used.
- ***RKA*** *(rough set-based knowledge acquisition):* Integrated set of AI and mathematical techniques, comprising discretization of continuous values attributes to discrete values, *reducts* generation (RG) for selecting essential attributes and LEM2 algorithm [34] for rules extraction.
- ***KBs*** *(knowledge bases):* Repositories of rough set rules, to predict T1DM and T2DM, and guideline rules, to identify abnormal observations and predict future trends. The rules are represented as production rules.
- ***HRBR*** *(hybrid rule-based reasoning):* Rule-based reasoning methodology that implements rough set rules for prediction of T1DM and T2DM and reference range reasoning that implements guidelines rules for finding abnormal observations.
- ***CTA*** *(correlation-based trend analysis*): a set of statistical methods, such as regression analysis and trend analysis to identify abnormal observations and predict future trends for prognosis service.

The operating mode of the proposed model has two stages, offline phase and online phase. The offline phase is focused on data preparation (structuring) from external knowledge sources that are presented in unstructured clinical charts and online diabetes guidelines (*i.e.*, PCG) and acquiring knowledge from these sources. The knowledge acquisition composed of manual and automatic procedures. In the manual process, first, patients’ clinical charts are transformed to structured form called diabetes mellitus information system (DMIS (RST uses a formalism that represents and analyses data in its specific format that is described in a structured form called information system, therefore we named our dataset as DMIS)) and guidelines to abnormalities and trend analysis rules (ATAR). In automatic acquisition, a set of rough set-based knowledge acquisition techniques are used to mine DM prediction rules (DMPR) from the DMIS. The rules are stored in knowledge bases that are used in the online process. The online phase is the live or execution phase of the model that delivers prediction and management services to physicians for supporting them in their decisions. This phase is activated by the arrival of a patient either a new patient for diagnosis or a registered one for follow-up. In the case that the patient is registered for the first time, HRBR methodology is triggered. In HRBR, rough set reasoning (RSR) diagnoses and predicts type of diabetes and in reference range reasoning (3R), abnormal observations are predicted. In the case that a patient is already registered, only the CTA part of the online phase is activated. CTA provides facility to physicians to see all the previous encounters of a patient in consolidated form and identify abnormal patterns out of them. It also provides analysis of future trends for all the observation and supports them to find any potential future risk. Hence, the physician can take preventive measures.

## 3. Methodology

Complete concept of the proposed H2RM for the prediction and management of diabetes is discussed step-by-step in this section.

### 3.1. Patient Charts and Online Guidelines

**Figure 2:**
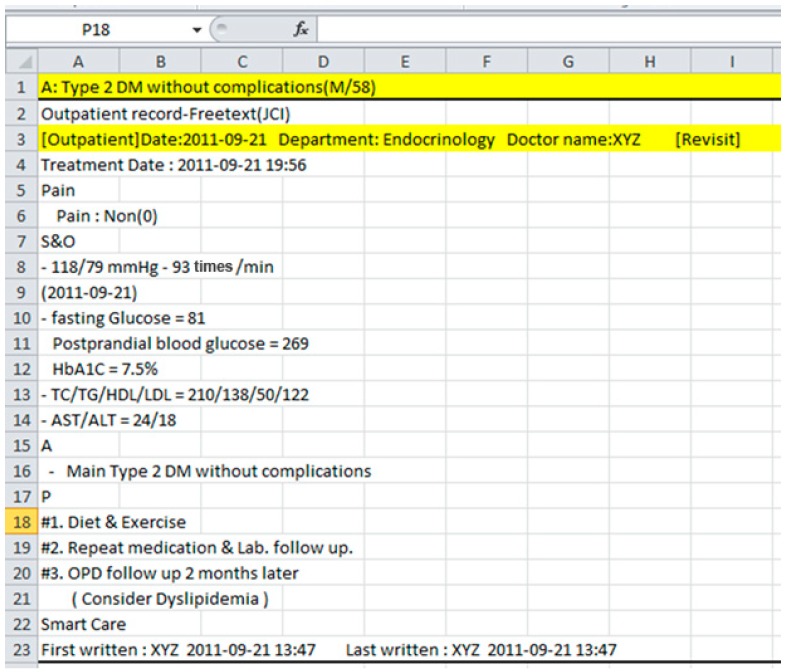
An encounter of type-2 diabetes mellitus patient (T2DM), following subjective, objective, assessment, and plan (SOAP)-based protocol.

Data from 50 diabetes patients, 20 with type-1 and 30 type-2 DM is acquired from a local hospital that records patients observations in clinical charts, following SOAP (Subjective, Objective, Assessment and Plan)-based protocol [35]. In the hospital, data are collected over the period of four years from 2008 to 2011 with an average of eight encounters per patient. The minimum number of encounters recorded for a patient is two and the maximum is eighteen. In the charts, patient information containing physiological data, clinical laboratory tests findings, diagnosis information and recommendations are recorded in *Subjectivity*, *Objectivity*, *Assessment* and *Planning* sections. In all the charts, Subjectivity and Objectivity sections are merged in one section, titled S & O. The *Assessment* section is put at the top of each encounter and sometime before the *Planning* section. Different encounters with the same patients are recorded in the same chart to maintain their history in one document. An example of an encounter with a T2DM patient’s chart is shown in Figure 2.

There are a number of inconsistencies in the charts, such as naming variations, incomplete values, miss-placement of observations, *etc*.

Similarly, to assist physician in automatic abnormalities identification in observations and predicting future trends, online diabetes guidelines are identified for rules creation. The most important predictors in diabetes prediction are body mass index (BMI), blood pressure, fasting blood glucose, glycated hemoglobin (HbA1c), lipids, and liver function tests (LFT), therefore online guidelines associated with these predictors are searched with domain experts support. These are listed in Table 1.

**Table 1:** List of guidelines used for managing diabetes mellitus.

S.No	Predictor	Guidelines	References
1	BMI	WHO: BMI classification	WHO [36]
2	BP: SBP, DBP	JNC 7 report, AHA	JNC [37,38,39]
3	FBS	American Diabetes Association. Diabetes Care	ADA [40,41]
4	HBA1c	American Diabetes Association, NICE	ADA [40], NICE [42,43]
5	Lipids: TC, TG, HDL, LDL	NCEP, ADA	NCEP [44], ADA [45]
6	LFT: ALT, AST	Liver disease (LD), Mayo Clinic	LD [46], Mayo Clinic [47]

### 3.2. Charts and Guidelines Translation

**Figure 3:**
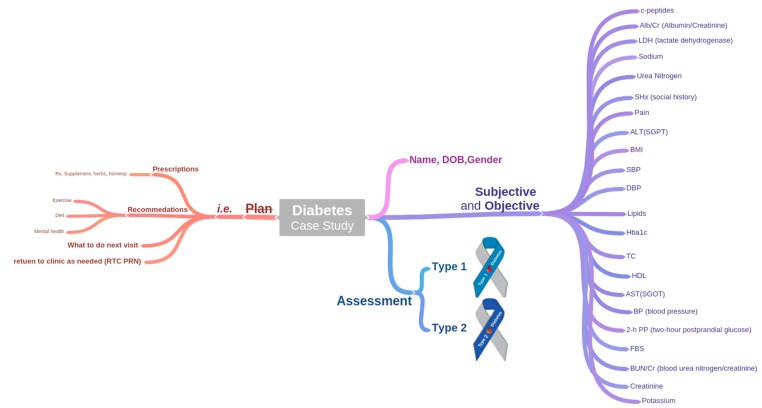
Distribution of diabetes patient’s observations in subjective, objective, assessment, and plan (SOAP)-based clinical chart.

We have carefully analyzed the unstructured charts and manually parsed all the observations to transform into structured format. The list of observations, extracted from charts is shown in the right-hand side of Figure 3. Since we have restricted this study only to prediction and management of diabetes in terms of abnormalities identification and trend analysis, the plan part of chart is not considered.

A structured schema is created for the observations recorded in the clinical charts. The schema records the following observations: (PID) patient identifier, encounter (ID) identifier, height, weight, waist, BMI, FHx (family history), SHx (social history), Gender, Age, TDM (type of diabetes mellitus), Complication, Pain, BP (blood pressure), Symptoms, 2-H PP (two-hour postprandial glucose), FBS (fasting blood glucose), Hba1c (glycosylated hemoglobin), Diabetes History, Hypoglycemia, Lipids, BUN/Cr (blood urea nitrogen/creatinine), AST/ALT (aspartate transaminase/alanine transaminase), Urea Nitrogen, Creatinine, Sodium, Potassium, LDH (lactate dehydrogenase), Alb/Cr (Albumin/Creatinine), and c-peptides for each patient. Each encounter is translated to a record in the schema (*i.e.*, DMIS). For each patient, all encounters are parsed and added into the dataset. In total there are 391 recorded encounters, with a distribution of 113 for T1DM and 278 for T2DM.

Since a number of attributes have incomplete values, they are dropped from DMIS. The criterion used is that the attributes with missing values ≥20% most likely produce miss-leading results, therefore they are filtered out and dropped. Similarly, we have split BP attribute to SBP (systolic blood pressure) and DBP (diastolic blood pressure) and Lipids into its four constituents: TC (Total cholesterol), TG (Triglycerides), HDL (High-density lipoprotein), and LDL (Low-density lipoprotein). Liver function tests, AST/ALT are split into AST and ALT. The final output of SOAP-based charts translation is a computer processable dataset, *i.e.*, DMIS.

Similarly, the guidelines listed in Table 1 are translated to simple reference rules that define normal and abnormal reference ranges of values for BMI, blood pressure, glucose, glycosylated hemoglobin, lipids, AST, and ALT attributes. These are shown in Table 2a–k.

After creation of these rules, they are stored in knowledge base under the abnormal and trend analysis rules repository (ATAR). These rules are used in the live execution process of H2RM.

**Table 2:** Rules defined for management of diabetics observations, based on reference ranges, extracted from guidelines (Table 1).

**(a) BMI**		**(b) TC**	
**Interval (Condition)**	**Interpretation (Decision)**	**Interval (Condition)**	**Interpretation (Decision)**
(−∞, 18.5)	underweight	(−∞, 200)	desirable
[18.5, 24.9]	normal	[200, 239]	borderline high
[25, 30)	overweight	[240, ∞)	high
[30, ∞)	obese		
**(c) SBP**		**(d) TG**	
**Interval (Condition)**	**Interpretation (Decision)**	**Interval (Condition)**	**Interpretation (Decision)**
(−∞, 120)	normal	(−∞, 150)	normal
[120, 139]	prehypertension	[150, 199]	borderline-high
[140, 159]	hypertension stage 1	[200, 499]	high
[160, 180]	hypertension stage 2	[500, ∞)	very high
[181, ∞)	hypertensive crisis		
**(e) DBP**		**(f) LDL**	
**Interval (Condition)**	**Interpretation (Decision)**	**Interval (Condition)**	**Interpretation (Decision)**
(−∞, 80)	normal	(−∞, 100)	optimal
[80, 89]	prehypertension	[100, 129]	near or above optimal
[90, 99]	hypertension stage 1	(129, 159]	borderline high
[100, 110]	hypertension stage 2	(159, 189]	high
(110, ∞)	hypertensive crisis	(189, ∞)	very high
**(g) FBS**		**(h) HDL**	
**Interval (Condition)**	**Interpretation (Decision)**	**Interval (Condition)**	**Interpretation (Decision)**
(−∞, 70)	hypoglycemia	(−∞, 40)	low
[70, 99]	normal	[40, 60)	normal
(99, 126]	pre-diabetic	[60, ∞)	high
(126, ∞)	diabetic		
**(i) HbA1c**		**(j) AST (SGOT)**	
**Interval (Condition)**	**Interpretation (Decision)**	**Interval (Condition)**	**Interpretation (Decision)**
[4, 5.9]	hypoglycemia	(−∞, 5)	low
(5.9, 6.4]	prediabetes	[5, 40]	normal
(6.4, 7.4]	diabetes	(40, ∞)	high
(7.4, ∞)	diabetes with Higher risk		
**(k) ALT (SGPT)**			
**Interval (Condition)**	**Interpretation (Decision)**		
(−∞, 7)	low		
[7, 56]	normal		
[57, ∞)	high		

Legend: “[”or “]” means inclusive, “(” or “)” means exclusive, “∞” means ± infinity.

### 3.3. Rough Set-Based Knowledge Acquisition

The translated diabetes dataset, DMIS, contains 391 instances for T1DM and T2DM as the training data for mining prediction rules to predict diabetes for new patients. Generally, clinical datasets are high dimensional [48] and usually contains incomplete values [49], which physicians either consider default values or less essential to be recorded. This makes the data inconsistent and vague in nature. To cope with these situations, we adopt a well-known RST [11,12], initially proposed by Pawlak. We mine prediction rules from the diabetes data using techniques supported by RST. Our choice of RST is due to its powerful nature of analyzing and handling vague and uncertain information in classification problems [50]. RST uses a formalism that represents and analyses data in its specific format that is described in a structured form called information system, therefore we named our dataset DMIS. The lower and upper approximations concepts of RST help to solve the problems of data vagueness, uncertainty, and incompleteness in class definition [51]. The definitions of lower and upper approximations are based on equivalence relations [52]. Approximation helps in partitioning the dataset into positive, negative and boundary regions, which ultimately help in solving the problems of vagueness and inconsistencies. The proposed RKA model includes the following phases, such as preprocessing, data reduction and rules creation. These phases work in a sequential flow, as shown in Figure 1.

#### 3.3.1. Preprocessing Phase

In the dataset, sometimes attributes contain redundant information which need to be filter out using expert knowledge to get the list of essential attributes. In our case, we first use expert knowledge to pre-select essential attributes for rough set information system. For example, the calculated attribute BMI is selected and its ingredients, height and weight, are dropped to avoid duplications. Similarly, the attributes “past history” and pain are dropped because their values are usually same throughout the dataset. Apart from the expert-based pre-selection method, we use three criteria to treat missing values in our dataset. These include, attributes dropping, average/frequent value and immediate previous/next value. Table 3 summarizes these strategies along with their criteria and scope. In the diabetes dataset, FHx and SHx are dropped, based on our first criteria. For these attributes, the proportion of missing values is greater than 20% of the whole dataset; therefore, they are dropped. The list of essential attributes, obtained after applying experts knowledge and attribute dropping criteria, includes: Gender, Age, BMI, SBP, DBP, FBS, Hba1c, TC, TG, HDL, LDL, AST, ALT and TDM.

These attributes contain incomplete values for the observations SBP, DBP, FBS, Hba1c, TC, TG, HDL, LDL, AST, ALT with 6%, 6%, 4%, 1%, 9%, 9%, 9%, 15%, 19% and 19% ratio, respectively. In these cases, the patient level criteria become valid and their corresponding strategies are applied. The description of these criteria and the associated strategies are described in Table 3. The average and frequent values strategies are the most frequently used techniques that are applied to numeric and nominal value attributes [53]. We performed experiments for these strategies in Rapid Miner environment [54]. Similarly, if only two values are missing in an attribute of the encounters of a patient then immediate previous/next value strategy is used. In this case, either *E_n−_*_1_ or *E_n+_*_1_ encounter value is used, depending on the position of missing value that either appears in consecutive or non-consecutive encounters. If values are missing in two consecutive encounters, one is filled with *E_n−_*_1_ and the other with *E_n+_*_1_. The rational of this strategy is that physicians usually do not record values if they see no change in the observation of a patient. Therefore, either preceding or proceeding value can be the best candidate for the missing value. A working example of one patient encounters with missing values and filled values is given in Table A1, Appendix A.1.

**Table 3:** Missing value treatment, criteria and strategies, applied to the diabetes mellitus dataset.

Scope	Criteria	Strategy
Dataset level (whole population)	If any attribute of the dataset has missing values in 20% or more than 20% records of the whole dataset	Drop the attributes from the dataset, this may leads to incorrect results
Patient level (whole encounters of one patient)	If any attribute has missing values in 2 or less than 2 encounters of a patient	Use immediate previous/next encounter’s values of the same patient Immediate previous/next encounter value, if missing values are non-consecutive Immediate previous encounter value for the first missing value and immediate next value for the second missing value, if missing values are consecutive
	If any attribute has missing values in less than 20% of the encounters of a patient	Use average/frequent value strategy within encounters of the same patient Compute average of all the values of that attribute for the same patient, if attribute is numeric Compute frequent value within all the encounters of the same patient, if the attribute is nominal
	If any attribute has missing values in more than 20% of the encounters of a patient	Use average/frequent value strategy within patients of the same class Compute average of all the values of all the patients in the same class, if attribute is numeric Compute frequent value within all the patients of the same class, if the attribute is nominal

The final preprocessed dataset, with filtered attributes and filled missing values, has the following clinical characteristics, summarized in Table 4.

**Table 4:** Clinical characteristics of the diabetes patients.

Characteristic	Average	Min. Value	Max. Value	Std. Deviation
BMI	23.0	16.2	32.0	3.2
Gender	M (256), F (135)			
Age	48.8	20.0	85.0	15.4
SBP	120.8	89.0	190.0	14.9
DBP	74.5	45.0	115.0	10.2
FBS	137.6	49.0	394.0	43.9
Hba1c	8.0	4.2	14.6	2.0
TC	169.5	0.0	371.0	37.7
TG	101.0	18.0	634.0	80.9
HDL	64.5	31.0	196.0	23.7
LDL	82.2	15.0	180.0	29.4
AST (SGOT)	22.0	11.0	65.0	7.8
ALT (SGPT)	26.6	8.0	120.0	18.0
TDM	T2DM (278), T1DM (113)			

#### 3.3.2. Data Reduction Phase

Clinical data have continuous values that randomly vary. If these values are used in its original form for mining rules, then rough set will extract huge number of rules, which are intractable [55]. Therefore, all continuous values attributes (e.g., except gender and TDM) first need to be reduced to finite number of intervals [56] and then use in rule mining process. Traditional rough set theory uses different types of discretization methods [56], which define discrete intervals without taking domain knowledge into account. These methods use statistical, entropy, genetic algorithms, fuzzy set theory and Boolean reasoning approaches to split continuous values into discrete intervals [56]. However, none of these methods use semantics of the values of attributes. In the healthcare domain and service generation, semantics of medical data values have significant importance. For example, in the case of our diabetes dataset, continuous values of the SBP attribute (measured in mm Hg) give information that the patient is either in normal (<120), prehypertension (120–139), hypertension stage 1 (140–159), hypertension stage 2 (160–180), or hypertensive crisis (≥181) status. Here, it is very important to discretize the continuous values of SBP in a way to retain their semantic categories in the discretized range/interval. If not, then the rules mined, based on these discretized values, will not reflect the correct range or interval of the value. The exiting discretization approaches do not care about such semantics. For example, if we use the well-known Boolean reasoning approach [57], it gives only three intervals for the same SBP attribute in our dataset. These are, (SBP < 110), (SBP 110–116), (SBP ≥ 117), which do not reflect the real semantic categories of SBP. To overcome this problem, we propose a semantic interval-based discretization scheme that consumes domain knowledge for discretizing continuous values. In the scheme, we first define cut points for discretization using standard reference ranges for each attribute, as shown in Table 2. This knowledge makes the intervals and cut-points more meaningful from clinical perspective and results in meaningful rules. The set of cut-points, their corresponding intervals, and the discrete value for each attribute are shown in Table 5.

**Table 5:** Set of cut-points and corresponding intervals for discretization of the Diabetes Mellitus Information System (DMIS).

Attributes	# Cut-Points: Cut-Points Description	# Intervals: Interval Description	Discrete Value for Interval	Guidelines
BMI	3: 18.5; 25; 30	4: (−∞, 18.5), [18.5, 24.9], [25, 30), [30, ∞)	0, 1, 2, 3	WHO [36]
Gender	NA	NA	NA	-
Age	2: 30; 50	3: (−∞, 30), [30, 50], (50, ∞)	0, 1, 2	-
SBP	4: 120; 140; 160; 181	5: (−∞, 120), [120, 139], [140, 159], [160, 180], [181, ∞)	0, 1, 2, 3, 4	JNC 7 report, AHA [37,38,39]
DBP	4: 80; 90; 100; 110	5: (−∞, 80), [80, 89], [90, 99], [100, 110], (110, ∞)	0, 1, 2, 3, 4	JNC 7 report, AHA [37,38,39]
FBS	3: 70; 99; 126	4: (−∞, 70), [70, 99], (99, 126], (126, ∞)	0, 1, 2, 3	ADA [40,41]
Hba1c	3: 5.9; 6.4; 7.4	4: [4, 5.9], (5.9, 6.4], (6.4, 7.4], (7.4, ∞)	0, 1, 2, 3	ADA [40], NICE [42,43]
TC	2: 200; 240	3: (−∞, 200), [200, 239], [240, ∞)	0, 1, 2	NCPE [44], ADA [45]
TG	3: 150; 200; 500	4: (−∞, 150), [150, 199], [200, 499], [500, ∞)	0, 1, 2, 3	NCEP [44], ADA [45]
HDL	2: 40; 60	3: (−∞, 40), [40, 60), [60, ∞)	0, 1, 2	NCEP [44], ADA [45]
LDL	4: 100; 129; 159; 189	5: (−∞, 100), [100, 129], (129, 159], (159, 189], (189, ∞)	0, 1, 2, 3, 4	NCEP [44], ADA [45]
AST(SGOT)	2: 5; 40	3: (−∞, 5), [5, 40], (40, ∞)	0, 1, 2	LD[46], Mayo Clinic [47]
ALT(SGPT)	2: 7; 57	3: (−∞, 7), [7, 56], [57, ∞)	0, 1, 2	LD[46], Mayo Clinic [47]

Legend: “[”or “]” means inclusive, “(”or “)” means exclusive, “∞” means ± infinity.

After applying discretization process based on the cut-points, we obtained discretized information system (DIS). A partial view of the discretized DIS is presented in Table 6.

The discretized output is used in rough set data exploration system (ROSE 2) [58,59] for further processing. ROSE 2 system operates on discrete value format of the continuous values, shown in fourth column of Table 5. Setup of this tool for processing discretized data is shown in Appendix A.2.

After attributes values reduction using discretization, the next step is to create *reducts*, which are feature subsets of attributes in the original information system (*i.e.*, DMIS). *Reducts* facilitate in the process of rule mining and classifying same dataset with same accuracy [11,12,60]. We adopted *lattice reduct search* method, implemented in ROSE 2 system [58,59] with the default configuration. The set of all possible *reducts* obtained are shown in Table 7.

In all the *reducts*, total number of participating attributes are 12 and only one attributes TC is not considered in either of the *reduct*. The frequency of attributes Gender, DBP, TG and OT in all the *reducts* is 50% while the rest of attributes have 100% participation, which means that they appear in all *reducts* and are therefore essential.

Like *reduct*, *core* is another important concept of RST, which comprises only the most relevant attributes in the original information system. If any attribute is removed from the *core*, the accuracy of classification rules drastically dropdown, therefore we apply the *core* generation operation in ROSE 2 to get the final key attributes. *Core* is generated using intersection operation over all *reducts*. In our case, the *core* consists of the features shown in Equation (1)

Core(DIS) = Intersection (RED(DIS)) = {BMI, Age, SBP, FBS, Hba1c, HDL, LDL, PT}
(1)

Prediction accuracy of the original set of attributes and the *core* attributes was measured. The objective of measuring accuracy is to show effectiveness of the reduced attributes and overall attributes in the original dataset. When measured, *core* attributes produced 0.9744% accuracy, while the all 13 attributes of the original information system produced 0.9872% accuracy. The total reduction in accuracy is only 0.0138%, which is almost negligible. However, the *reduct* and *core* operations of RST reduced the number of attributes by more than one third, which reduce the complexity of building the prediction model. Appendix A.3 shows a working example of our diabetes dataset for generation of *reduct* and *core* in ROSE 2 environment.

**Table 6:** Partial data of diabetes mellitus Information System in interval format after discretization.

DiscBMI	Gender	DiscAge	DiscSBP	DiscDBP	DiscFBS	DiscHba1c	DiscTC	DiscTG	DiscHDL	DiscLDL	DiscAST	DiscALT	TDM
[18.5, 24.9]	M	(50, ∞)	[120, 139]	(−∞, 80)	(99, 126]	(7.4, ∞)	(−∞, 200)	(−∞, 150)	(−∞, 40)	(−∞, 100)	[5, 40]	[7, 56]	T2DM
[25, 30)	M	[30, 50]	[140, 159]	[100, 110]	[70, 99]	(7.4, ∞)	(−∞, 200)	[150, 199]	[40, 60)	(−∞, 100)	[5, 40]	[7, 56]	T1DM
[18.5, 24.9]	F	(50, ∞)	(−∞, 120)	(−∞, 80)	(126, ∞)	(6.4, 7.4]	[200, 239]	(−∞, 150)	[40, 60)	[100, 129]	[5, 40]	[7, 56]	T2DM
.	.	.	.	.	.	.	.	.	.	.	.	.	.
.	.	.	.	.	.	.	.	.	.	.	.	.	.
(−∞, 18.5)	F	[30, 50]	(−∞, 120)	(−∞, 80)	(126, ∞)	(7.4, ∞)	(−∞, 200)	(−∞, 150)	[60, ∞)	(−∞, 100)	[5, 40]	[7, 56]	T1DM
[25, 30)	F	(50, ∞)	(−∞, 120)	[80, 89]	(99, 126]	(5.9, 6.4]	(−∞, 200)	(−∞, 150)	[60, ∞)	(−∞, 100)	[5, 40]	[57, ∞)	T2DM

Legend: “[”or “]” means inclusive, “(” or “)” means exclusive, “∞” means ± infinity.

**Table 7:** List of all possible *reducts* for the Discretized Information System after applying Lattice Reduct Search method.

Reduct #	# Attributes	Reduct (Attributes)
1	10	{BMI, Gender, Age, SBP, DBP, FBS, Hba1c, HDL, LDL, PT}
2	10	{BMI, Age, SBP, DBP, FBS, Hba1c, TG, HDL, LDL, PT}
3	10	{BMI, Gender, Age, SBP, FBS, Hba1c, HDL, LDL, OT, PT}
4	10	{BMI, Age, SBP, FBS, Hba1c, TG, HDL, LDL, OT, PT}

#### 3.3.3. Rules Mining and Validation Phase

Once the *core* attributes are selected, the next step is to mine decision rules from the discretized information system for the *core* attributes using learning from example module, version 2 (LEM2) algorithm [34]. We have used the *basic minimal covering* criteria of LEM2 algorithm implemented in ROSE 2 system [59]. The DIS (setup, results of the experiments performed in ROSE 2 system, and data in anonymized form can be provided on reader’s personal request) contains 391 instances that are used for mining rules to predict diabetes types. In total, 23 rules are mined. One rule is approximate, with inconsistent prediction for the same condition attributes. Extracted partial rule set is shown in Table 8.

**Table 8:** A Partial list of rules extracted from discretized information system (DIS) using rough set (RS) learning from example module, version 2 (LEM2) algorithm.

Rule #	Prediction for TDM	Prediction Rule	Significance
1	(T1DM)	(BMI = [18.5, 24.9]) and (Age = (50, ∞)) and (SBP = [120, 139]) and (Hba1c = (7.4, ∞)) and (TC = (−∞, 200)) and (SGPT = [7, 56])	20 (17.70%)
2	(T2DM)	(Gender = M) and (SBP = (−∞, 120)) and (Hba1c = (6.4, 7.4]) and (LDL = [100, 129])	17 (6.12%)
3	(T2DM)	(BMI = [18.5, 24.9]) and (Age = [30, 50]) and (SBP = (−∞, 120)) and (TG = (−∞, 150)) and (HDL = [40, 60))	23 (8.27%)
4	(T1DM)	(SBP = [120, 139]) and (DBP = [80, 89]) and (Hba1c = (5.9, 6.4]) and (HDL = [40, 60)) and (SGPT = [7, 56])	7 (6.19%)
5 (approximate rule)	(T1DM) OR (T2DM)	(BMI = [18.5, 24.9]) and (Age = (50, ∞)) and (FBS = 3) and (Hba1c = (126, ∞)) and (TG = (−∞, 150)) and (LDL = (−∞, 100)) and (SGPT = [7, 56])	[5, 5] [2, 3]

Legend: “[”or “]” means inclusive, “(” or “)” means exclusive, “∞” means ± infinity.

Table 8 shows decision attribute of the rule, ingredients of the rules (*i.e.*, condition attributes with values) and significance value in columns 2–4, respectively. The significance describes coverage of the rule its own class. For example, rule 1 has 17.7% significance value in its class T1DM that supports 20 instances of the training information system. After creation of the rules, the prediction model is stored in the knowledge base within DM prediction rules repository, DMPR. These rules are used in the live execution process of H2RM. Details of the experimental setup of ROSE 2 system for the rule mining process is given in Appendix A.4.

Validation of the prediction model (rules extracted using rough set LEM2 method [34]) is performed using 10-fold cross validation approach. The details are given in Section 4 and the setup of the ROSE 2 system is described in Appendix A.5.

### 3.4. Hybrid Rule-Based Reasoning

Online phase of the proposed H2RM is based on RBR methodology, which internally uses two levels of reasoning in sequential way. In the first level, rough set-based reasoning (RSR) methodology is activated for those patients who are not registered before. In this process, the RSR engine loads rules from the DMPR repository and executes them on the current observations of the patient. Diabetes type is predicted from the patient’s observations and withheld till the second level of reasoning process is not completed. In the second level, reference range-based reasoning (3R) is performed over BMI, SBP, DBP, FBS, Hba1c, TC, TG, HDL, LDL, AST (SGOT), and ALT (SGPT) using reference range rules defined in Table 2 to categorize the observations as either normal, borderline, abnormal, risky, *etc*. This automatic categorization of the observations further assist physician in easy understanding and quick decision-making. The final results of HRBR are provided to physicians to assist them in diagnosis and analysis of the patient’s current observations. This process is shown in detail in Algorithm 1. **Algorithm 1** Hybrid Rule-based Reasoning (HRBR)**Input**: KB: Knowledge Base, E: Encounter**Output**: TDM, INTERPRETATION**Begin**ApplyHRBR (E), where {E|E is EncounterOfNonRegisterdUser,E:={Pid,OBS}}, OBS:={BMI, SBP, DBP, FBS, Hba1c, TC, TG, HDL, LDL, AST(SGOT), ALT(SGPT)}A.PerformRSR(E)
**// Rough Set Reasoning**  [Load Prediction Rules From Knowledge Base]1. DMPR:= LoadRulesFromKB(RULES that contain TDM as CONC);where CONC:={ T1DM, T2DM}2. [Execute Rules For Predicting Types of Diabetes] Foreach RULE in DMPR
Foreach CA in RULE //CA:={BMI, Age, SBP, FBS, Hba1c, HDL, LDL, PT}If CA.values ≠ E.OBS.value  THEN Try next RULEEndIfTDM:=CONC of the RULE;Goto Step BEndFor  EndFor 3. TDM=Message("UNDEFINED")B.Perform3R (E)
**// Reference Range-based Reasoning**  [Load Reference Range Rules From Knowledge Base]4. ATAR:= LoadRulesFromKB(RULES that contain INTERPRETATION as CONC);where CONC:={ Table 2.INTERPRETATION.Value}
[Execute Rules For Finding Current Status of Each Observation]5. Foreach RULE in ATAR
Foreach CA in RULE //CA:={BMI, SBP, DBP, FBS, Hba1c, TC, TG, HDL, LDL, AST(SGOT), ALT(SGPT)}If CA.values ≠ E.OBS.value THEN Try next RULEEndIfINTERPRETATION []:=CONC of the RULE;EndFor   EndFor C. PHYSICIAN∶=ProvideResults (Pid,TDM, INTERPRETATION)**End**

Algorithm 1 has four main functions. These are defined for activation of HRBR, rough set-based reasoning, reference range-based reasoning and final results propagation. When a new patient arrives in the hospital and his observations are recorded, the main function of HRBR, ApplyHRBR (), is activated. This function, called the rough set-based reasoning function, PerformRSR(), for predicting diabetes type. The process of rough set reasoning starts with loading rules from knowledge base using the function, LoadRulesFromKB(). Once rules are loaded, execution of rules starts and final decision is obtained either as T1DM, T2DM or UNDEFINED.

After diabetes prediction, physicians are usually interested in knowing the exact status of the observations of the patient. For this purpose, reference range-based reasoning is activated using the function, Perform3R (). Like rough set reasoning, first, rules are loaded from knowledge base, and then they are executed one-by-one to find out whether the current value for that observation is normal, borderline, risky, *etc*. Finally, the function ProvideResult() propagates the results of rough set reasoner and reference range reasoned, in integrated from, to physician for further assessment and final decision.

### 3.5. Diabetes Management: Correlation-Based Trend Analysis for Prognosis

In the online phase of H2RM, when a registered patient visits hospital for follow-up and new observations are recorded then physician usually desires to review past history of all encounters of the patient. This is an essential step for them to further analyze the patient’s conditions and prescribe medications or provide general wellbeing recommendations or consult patient regarding the next follow-up, *etc.* Moreover, they are also interested in seeing future trends of the patient’s observations, based on the current and past observations, in order to predict future and take preventive measures. However, they are unable to get all these benefits in the current scheme of clinical charts, where the observations are inconsistent and placed randomly with different naming convention, *etc.* in excel sheets. The literature listed in this paper lack the capability of transforming these clinical charts to structured data format and building management and trend analysis services for physician to support them in decision-making. To overcome these shortcomings, and support physicians with comprehensive insights of the past observations of patients, we propose a correlation-based trend analysis technique.

**Figure 4:**
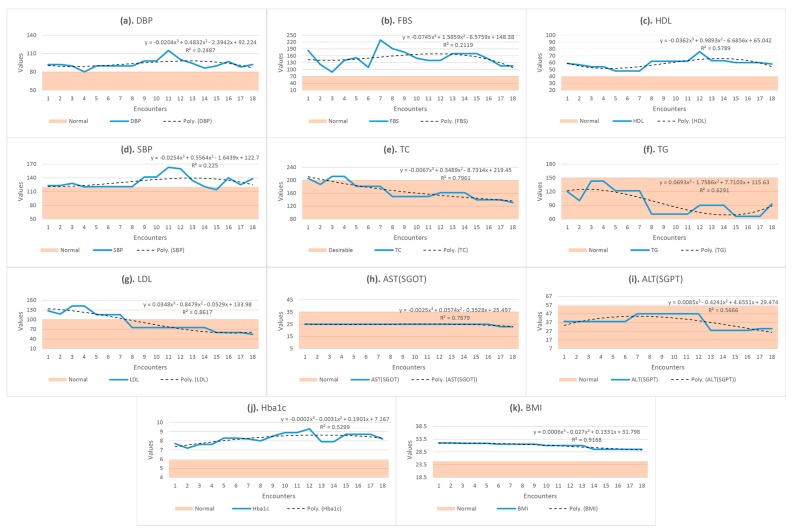
Correlation-based trend analysis for prognosis of diabetes mellitus. The bold-faced blue line represents scatterd line graph of the current observations, the dotted black line shows future polynomial trendline for future prediction and the light orange strap represents normal ranges of the observations.

Correlation analysis is one of the important future trends prediction technique applied to numeric data [61]. We adopt this technique in our study for analyzing abnormal trends in patient observations. For correlation and trend analysis, we used MS Excel [62] as our experimentation tool. In the knowledge execution flow of H2RM, when a registered patient visits the hospital for follow-up, his observations are recorded and scattered line graphs is drawn for the current and past observations, as shown in Figure 4a–k. It is represented by the bold-faced blue line in the graph. Furthermore, a correlation-based polynomial trendline of order 3 is added to the graph to predict future trend of the observation. We also compute residue *R^2^* value to the trendline to show accuracy of the future prediction for new encounters. The selection of polynomial trendline for future prediction is due to the fact that clinical values, always, gradually fluctuate rather than move sharply. Polynomial trendlines with order 3 have two peaks or bottom values in the regression equation.

The proposed CTA provides two insights to physicians: current status of the patient’s observation, whether normal, abnormal, *etc.* and future trends. Hence, the physician is assisted to see all the relevant information one place.

## 4. Experiments and Results

### 4.1. Evaluation Criteria

To evaluate the proposed hybrid RS reasoning model, a number of evaluation criteria can be used, such as prediction accuracy, precision, recall, F-measure, balanced accuracy and end user (physician in our case), satisfaction, *etc.* [63]. These criteria can be grouped into system-centric (focus on system accuracy, precision, recall, *etc.*) and user-centric (focus on user satisfaction, *etc.*) [64]. A good evaluation criterion can be the one taking both system centric and user centric parameters into account. However, in our evaluation, we stick to only the system centric approach due to the prototype implementation of H2RM. We use average accuracy and balanced accuracy evaluation metrics to evaluate the performance of our proposed model. The prediction rules derived by the rough set knowledge acquisition component are used to test data in the diabetes dataset and assess the performance.

### 4.2. Experimental Setup

H2RM consists of two main modules: offline knowledge acquisition and online knowledge execution. Therefore, we setup two sets of experiments. The first set is to mine prediction rules from the diabetes dataset and the second one is to provide real time services on top of these rules for new patient/encounter. For both sets, we used ROSE 2 system [58,59] in Windows environment in a PC with specification of Intel Pentium Dual-CoreTM (2.5 GHz) and RAM 4GB. For the first set of experiments, setup and detailed description is given in Section 3.3. The second set of experiments further consists of validation of mined rules and trend analysis of past and current encounters of a patient. Setup for the latter experiment is explained in Section 3.5, while for validation of mined rules, we use basic minimal covering technique of the RST with default parameters setting in ROSE 2 system. The default parameter settings are shown in Table 9 and a working example of the validation process is shown in Appendix A.5.

**Table 9:** Experimental setup used for validation of prediction rules in ROSE 2 system.

S.No	Parameters	Values
1	Test	k-fold cross validation
2	Number of passes	10
3	Majority threshold	21%
4	Minimum similarity	50%
5	Partially matched rules	All
6	Rule support	strength × similarity

### 4.3. Results

The results of first set of experiments are described in Section 3.3.3. In total, 23 rules are extracted from 391 instances of the dataset. Table 8 shows a partial list of the rules along with their significance values.

Results of the validation experiment are shown in Table 10.

**Table 10:** Confusion matrix (sum over 10 passes) describing overall output of the validation process.

Type of DM	T1DM	T2DM	None
T1DM	106 (TP)	7 (FN)	0
T2DM	9 (FP)	269 (TN)	0

Table 10 shows that 7/113 cases of T1DM are incorrectly predicted as T2DM and 9 T2DM cases are incorrectly predicted as T1DM. There is no such example, either of T1DM or T2DM, in which neither T1DM nor T2DM is predicted. Therefore, the “None” column is zero for both class. Average accuracy (%) of the prediction model and individual accuracies of each class (T1DM, T2DM) are shown in Table 11. The average predictive accuracy of the model is 95.91% with 4.09% incorrect predictions. Standard deviation of the percent incorrect predictions, for all the 10-folds of the model is 2.61, while for the individual classes are 6.16 and 4.11, respectively. The individual class level accuracy for class T1DM is 94.59% and for class T2DM is 96.85%.

**Table 11:** Average accuracy (%) of the model for individual class and overall model.

Type of DM	Correct	Incorrect	None
T1DM	94.59 ± 6.16	5.41 ± 6.16	0.00 ± 0.00
T2DM	96.85 ± 4.11	3.15 ± 4.11	0.00 ± 0.00
Total	95.91 ± 2.61	4.09 ± 2.61	0.00 ± 0.00

The results show that the predication accuracy for class T2DM is higher than the prediction accuracy of class T1DM. The reason for incorrect prediction of T1DM cases as T2DM and *vice versa* is due to the approximate rule (rule #23) of the prediction model.

**Figure 5:**
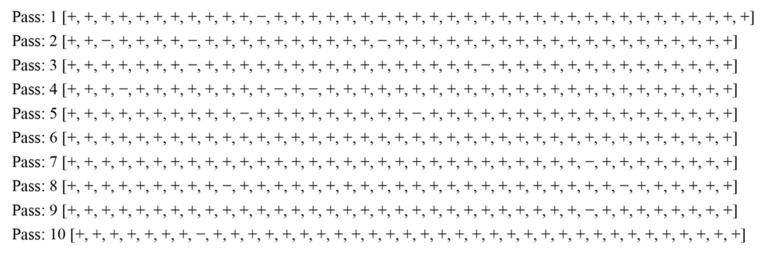
Test results of each pass of the 10-folds cross validation process.

To know results in terms of percent accuracy and percent error for each fold, we generate fold-wise test results. Figure 5 show the test results for each fold of the 10-fold cross validation process.

In Figure 5, plus sign (+) shows correct prediction, while negative sign (−) shows incorrect prediction. The first pass/fold contains 40 examples/instances while the rest include 39 instances each. Table 12a shows the percent accuracy and percent error of each pass of the 10-fold-testing process.

In Table 12b, we calculate average accuracy from the percent accuracy of each fold, which is 95.9%. In the same way, standard deviation is calculated from the percent error of each fold-test. Its value is 2.61.

The dataset we used for prediction and classification of diabetes as type-1 or type-2 has class distribution of 113:278. This shows that the ratio is greater than 1:2 for type-1 to type-2. Hence, type-2 is dominant over type-1. Therefore, to verify that our predictive model has produced unbiased results, measured as overall accuracy, we use the measure of balance accuracy, which is defined as arithmetic mean of sensitivity and specificity [65,66]. For computing balanced accuracy, we extract True Positive (TP), False Positive (FP), True Negative (TN) and False Negative (FN) evaluation measures from Table 10 and use Equation (2). Values of these measures are shown in Table 13.

(2) $$\text{Balanced accuracy}=\frac{0.5\times \text{TP}}{\text{TP}+\text{FN}}+\frac{0.5\times \text{TN}}{\text{TN}+\text{FP}}=0.9522$$

**Table 12:** Percent accuracy and percent error for each test of the 10-fold cross validation process along with average accuracy and standard error of all 10-folds

**(a) Percent Accuracy and Percent Error for Each Pass**					
**Pass Number**	**Fold Size**	**Incorrect Examples**	**Correct Examples**	**Percent Accuracy**	**Percent Error**
Pass 1	40	1	39	97.5	2.5
Pass 2	39	3	36	92.30769231	7.6923077
Pass 3	39	2	37	94.87179487	5.1282051
Pass 4	39	3	36	92.30769231	7.6923077
Pass 5	39	2	37	94.87179487	5.1282051
Pass 6	39	0	39	100	0
Pass 7	39	1	38	97.43589744	2.5641026
Pass 8	39	2	37	94.87179487	5.1282051
Pass 9	39	1	38	97.43589744	2.5641026
Pass 10	39	1	38	97.43589744	2.5641026
**(b) Average Accuracy and Standard Error for 10-Folds**					
No. Instances				391	
Total Number of Incorrect Examples				16	
Total Number of Correct Examples				375	
Average Accuracy				95.90384615	
Average Error				4.096153846	
Standard Error based on Percent Error of each Fold				2.61660764	
Average Accuracy ± Standard Errors				95.9 ± 2.6	

**Table 13:** Evaluation parameters for computing balanced accuracy.

True Positive (TP)	False Positive (FP)	True Negative (TN)	False Negative (FN)
106	9	269	7

The results of balanced accuracy (Equation (2)) and conventional average accuracy (Table 11 and Table 12) are the same, which shows that our predictive model performs equally well on either class (T1DM and T2DM).

The results of the final correlation-based future trend analysis experiment are shown in Figure 4. These results assist physicians in assessing patient observations from three perspectives: pattern of past and current observations (blue line graph), deviation of the observations from normal ranges (light orange strap), and prediction of future trend (dotted black line). A correlation equation of order 3, along with R^2^, show accuracy of future trend prediction for that observation.

## 5. Conclusions and Future Directions

Patient clinical charts and online guidelines are the most important available knowledge sources for physicians. A patient clinical chart data helps physicians stay aware of a patient’s present and past observations. They make future predictions regarding specific observations of the patient. In the above situation, physicians would like to have an intelligent prediction and forecasting system to automatically predicts a patient’s diabetes type and analyze future trends. Suitable prediction models and tools can help physicians to understand patient and make wise decisions.

This work designed a hybrid RS reasoning model, incorporating PCG as knowledge sources, CGT as translation and knowledge extraction process, RKA as rules mining process, KBs as knowledge repository, hybrid RBR as live prediction/classification process and CTA as future trend analysis of patients observations. The H2RM model first applies a manual process for clinical charts and guidelines translation, then uses semantic interval-based discretization, RS *reducts* generation and understandable decision rules extraction using the LEM2 algorithm [34] from the original diabetes dataset. Experimental results for the prediction model reveal that performance of the model is 95.91% in classifying diabetes types. Correlation-based trends analysis results suggest insights of the patient conditions to physicians in an appropriate way and assisting them in controlling risky stages.

Although the proposed hybrid model performs well, further experiments and improvements are required. Future studies should apply experiments to appropriately manage diabetics’ complications and risky behaviors, and provide support to physicians in automatic generation of treatments and wellbeing recommendations. Additionally, the proposed model can be applied to prediction problems in other fields. In the near future, we plan to implement this prototype model of the recommender system as a full working system and deploy in real setups. This will support physicians in real practices to support their diagnose decisions with the suggested decisions of the proposed diabetes recommender system. Furthermore, it will provide a base for new physicians to learn about diabetes from the decisions of the system.

## Figures and Tables

**Figure A1:**
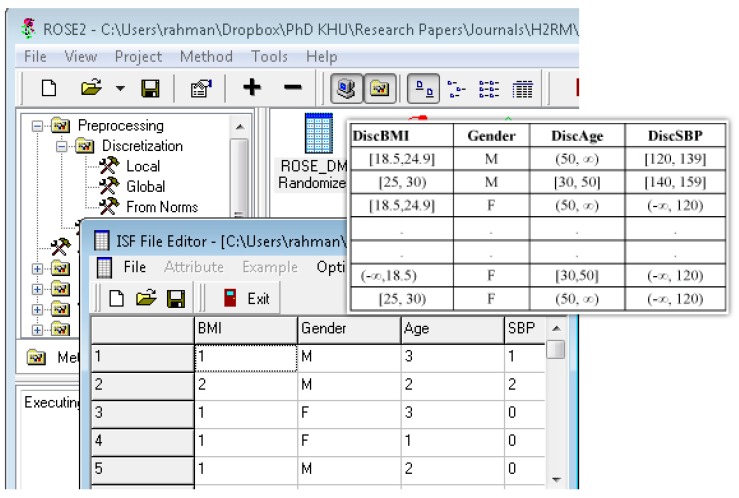
Final discretized results of the diabetes mellitus dataset.

**Figure A2:**
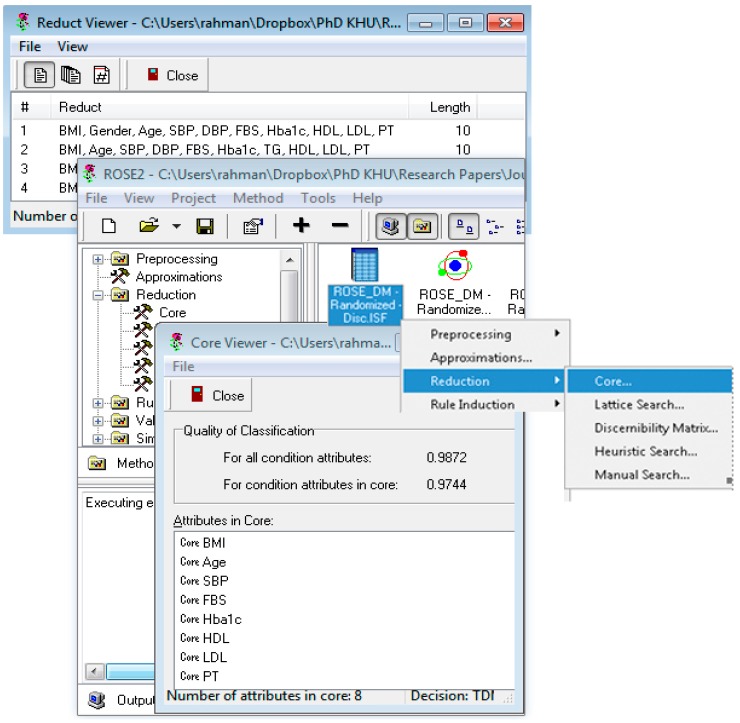
*Reducts* and *core* generation process in rough set data exploration system (ROSE 2) system along with the outputs.

**Figure A3:**
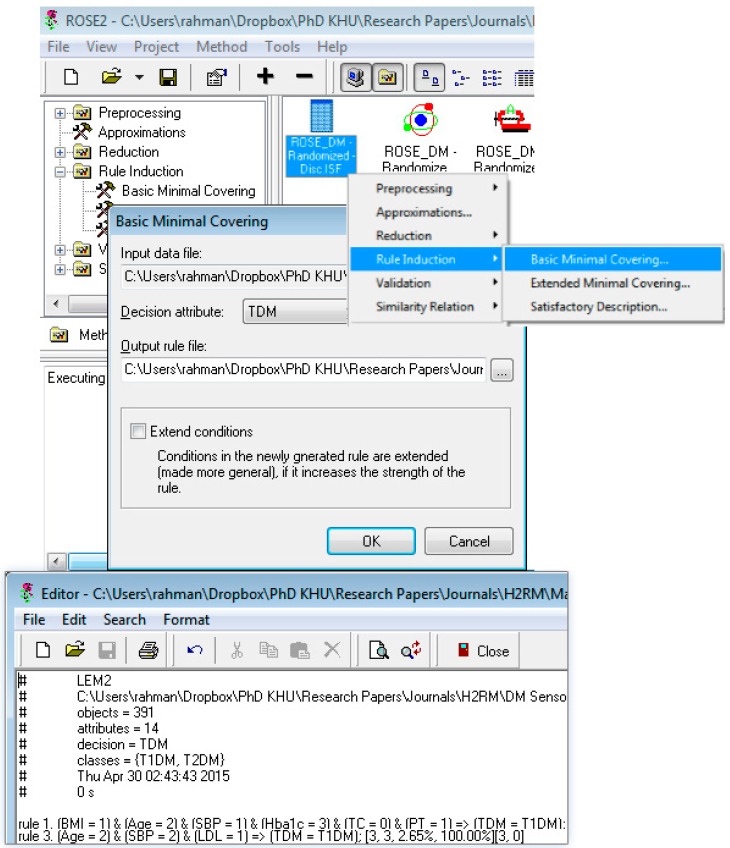
Rules induction setting in rough set data exploration system (ROSE 2) system along with the inducted rules as output.

**Figure A4:**
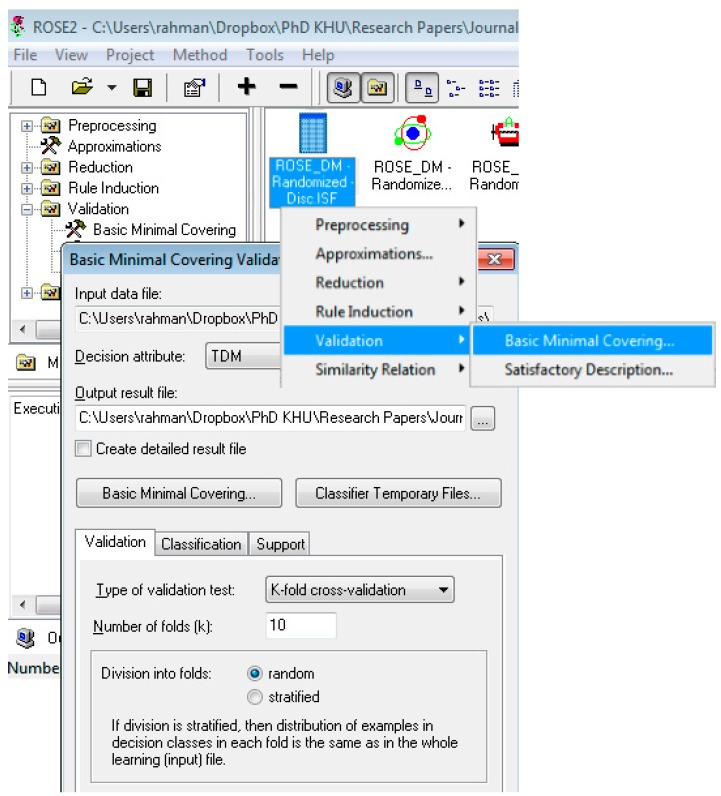
The validation process in the rough set data exploration system (ROSE 2) system for validating and testing mined rules.

## Appendix A

### A.1. Missing Values Treatment

Table A1a shows encounters of only one patient (PID = 50) with missing values in BMI, SBP, FHx, SHx, FBS Hba1c and LDL attributes. Table A1b shows the filled dataset after applying the missing value completion strategies, defined in the Preprocessing Section of the Methodology.

**Table A1:** Encounters of a single patient before and after applying the missing value completion strategies.

**(a) Encounters of Patient No. 50 with Missing Values in Encounters**												
**PID**	**EID**	**BMI**	**Age**	**SBP**	**FHx**	**SHx**	**FBS**	**Hba1c**	**HDL**	**LDL**	**ALT (SGPT)**	**TDM**
50	e1	21.1	41	109	no	yes	NULL	NULL	44	107	36	T2DM
50	e2	21.1	41	NULL	no	NULL	144	11.6	44	107	46	T2DM
50	e3	21.3	42	NULL	NULL	NULL	116	NULL	44	110	36	T2DM
50	e4	22.2	42	104	NULL	yes	155	6.6	42	150	64	T2DM
50	e5	NULL	42	123	NULL	NULL	NULL	NULL	42	NULL	64	T2DM
50	e6	22.1	42	123	NULL	NULL	246	8.9	52	165	39	T2DM
50	e7	22.1	42	114.0	no	NULL	240	7.2	50	130	40	T2DM
50	e8	22.2	42	191.0	NULL	NULL	230	9	51	162	45	T2DM
**(b) Filled Encounters after Applying Missing Value Treatment Strategies**												
**PID**	**EID**	**BMI**	**Age**	**SBP**	**FBS**	**Hba1c**	**HDL**	**LDL**	**ALT(SGPT)**	**TDM**		
50	e1	21.1	41	109	144	8.66	44	107	36	T2DM		
50	e2	21.1	41	109	144	11.6	44	107	46	T2DM		
50	e3	21.3	42	104	116	8.66	44	110	36	T2DM		
50	e4	22.2	42	104	155	6.6	42	150	64	T2DM		
50	e5	22.2	42	123	155	8.66	42	150	64	T2DM		
50	e6	22.1	42	123	246	8.9	52	165	39	T2DM		
50	e7	22.1	42	114.0	240	7.2	50	130	40	T2DM		
50	e8	22.2	42	191.0	230	9	51	162	45	T2DM		

### A.2. Discretization Setting in ROSE 2 System

Figure A1 shows the final discretized dataset in discrete value (*i.e.*, 0, 1, …) and range-value formats. ROSE 2 system operates on the discrete value format for further operations of *reduct* and *core* generation and rules induction. The range-value format is manually created for easy understanding and interpretation of the discrete values. The interpretation of both the formats can be seen in Table 5 in the Methodology Section.

### A.3. Reducts and Core Generation Settings in ROSE 2 System

Figure A2 shows how *reducts* and *core* are generated for a discretized dataset. First *reducts*, whose output is input for *core* operation, are generated.

### A.4. Rules Induction Setting in ROSE 2 System

Figure A3 shows how rules are mined from the *core* attributes using the *Basic Minimal Covering* method of the ROSE 2 system. This option use LEM2 [34] algorithm for mining rules.

### A.5. Rules Validation Setting in ROSE 2 System

Figure A4 shows default settings of ROSE 2 system for validating the rules generated in rule induction phase. The output is the average classification accuracy.
